# Mathematical Quantification of Transmission in Experiments: FMDV Transmission in Pigs Can Be Blocked by Vaccination and Separation

**DOI:** 10.3389/fvets.2020.540433

**Published:** 2020-11-20

**Authors:** Aldo Dekker, Herman J. W. van Roermund, Thomas J. Hagenaars, Phaedra L. Eblé, Mart C. M. de Jong

**Affiliations:** ^1^Wageningen Bioveterinary Research, Lelystad, Netherlands; ^2^Department of Quantitative Veterinary Epidemiology, Wageningen University, Wageningen, Netherlands

**Keywords:** foot-and-mouth, vaccine, transmission, reproduction ratio, pig, separation, disease control, epidemiology

## Abstract

Quantitative understanding of transmission with and without control measures is important for the control of infectious diseases because it helps to determine which of these measures (or combinations thereof) will be effective to reduce transmission. In this paper, the statistical methods used to estimate transmission parameters are explained. To show how these methods can be used we reviewed literature for papers describing foot-and-mouth disease virus (FMDV) transmission in pigs and we used the data to estimate transmission parameters. The analysis showed that FMDV transmits very well when pigs have direct contact. Transmission, however, is reduced when a physical barrier separates infected and susceptible non-vaccinated pigs. Vaccination of pigs can prevent infection when virus is administered by a single intradermal virus injection in the bulb of the heel, but it cannot prevent infection when pigs are directly exposed to either non-vaccinated or vaccinated FMDV infected pigs. Physical separation combined with vaccination is observed to block transmission. Vaccination and separation can make a significant difference in the estimated number of new infections per day. Experimental transmission studies show that the combined effect of vaccination and physical separation can significantly reduce transmission (R < 1), which is a very relevant result for the control of between-farm transmission.

## Introduction

Foot-and-mouth disease (FMD) is a contagious disease affecting cloven-hoofed animals and outbreaks can have major economic consequences. Due to the impacts of FMD, the German government decided in 1896 to finance FMD research which was led by Loeffler and Frosch (1). In the same year they started their research, they described FMD virus (FMDV) as an agent that passes bacterial filters (1), making FMDV the first animal virus ever described. In dairy cattle FMDV infection causes loss of milk production, in meat producing cattle and pigs, it reduces the feed conversion and in draft animals it reduces their availability for plowing and harvesting of crops. Furthermore, it can contribute to fertility problems, due to abortions and reduced conception rates, which will lead to a higher need of breeding animals (2). Control of FMDV has, in many countries, not only led to better economic results in livestock production but also opened new export markets resulting in increased sales of livestock products. Export of animals and animal products without limitations has, therefore, become very important for FMD free countries. An outbreak of FMD in an FMD free country will consequently not only have an impact on livestock production, but it will also have huge economic consequences due to closure of export markets. The economic losses caused by the 2001 FMD outbreaks in Europe and the repeated introduction of FMDV in South-Korea were enormous (3–6).

Since the presence of FMDV infection limits trade of animals and because of the success of national and regional campaigns in the past to control FMDV, the OIE (World Animal Health Organization) and FAO (Food and Agricultural Organization of the United Nations) have proposed to target FMDV for the next world-wide eradication after rinderpest (7). To improve the prospects for FMDV eradication and to be able to optimize control measures, it is necessary to have knowledge about and understand FMDV transmission. Outbreaks and the applied (different) control measures have been described in the past (8), but these studies did not quantify the effect of different control measures. A quantitative understanding of transmission and the contribution of different control measures in reduction of transmission is needed as input in mathematical models. Although quantitative data can be obtained during outbreaks (8–11), the accuracy of data obtained is limited and the effect of a single intervention cannot be studied. For this reason, experimental transmission studies for disease control are essential.

In studying (FMDV) infections, microbiologists have often tried to quantify certain parts of the transmission chain, e.g., the concentration of infectious particles in secretions and excretions that can contaminate the environment, and the probability of infection for various infection doses [the dose-response relationship (12)]. In principle, the full transmission chain can be simulated by combining these experimental quantifications by modeling the dissemination and dilution of the infectivity in the environment. However, this detailed type of modeling is subject to substantial uncertainties, and historical attempts to model FMD transmission in that way have overestimated the infection risks (13).

In contrast, using experimental observations to quantify the transmission rate parameter, which relates the fraction of susceptible and infectious individuals in a population to the hazard rate of a new infection occurring (14), seems to be an accurate way to estimate transmission and extrapolate to different situations (15, 16). The transmission rate parameter can also be expressed using the reproduction ratio (R) which is the average number of new infections caused by a typical (i.e., average) infected individual, during its whole infectious period in a fully susceptible population (i.e., a population only containing non-infected individuals. Please note that the population can also be non-infected vaccinated individuals if transmission in a vaccinated group is quantified). If R is below 1 only minor outbreaks can occur and the infection will eventually die off; when R is above 1 both minor and major outbreaks can occur (17, 18). The parameter R is determined not only by the average level of susceptibility in the population, but also by the infectivity of a typical infected animal, i.e., the average infectivity of the infectious animals in that population. It is, however, possible to quantify R without quantifying susceptibility and infectivity precisely.

FMD vaccine evaluation in cattle, sheep and pigs is extensively reviewed in Cox and Barnett (19). In pigs many experimental studies have been performed in the past (20–37). Most of these vaccine studies, however, were performed to demonstrate protection against clinical disease, protection against sub-clinical infection, to measure reduction of virus titres in excretions and secretions and/or measure the effect on immune responses. In some studies pigs were infected by injection. In several other experiments contact exposure to non-vaccinated seeder pigs was used, and clinical protection against challenge was studied early and late after vaccination (22–25, 32). In these experiments a short exposure period of 1, 2, and 4 h was used; in one experiment, where the aim was to infect several vaccinated pigs, a 9 h exposure period was used (32). But only a limited number of these experiments were designed to quantify how vaccination can reduce FMDV transmission.

To quantify the transmission rate parameter β (i.e., the average number of new infections caused by a typical infected individual, per unit of time in which the individual is infectious in a fully susceptible population) or reproduction ratio R (definition see above), an experimental design fitting those objectives is necessary (for estimation of β and/or R). In these experiments, infected “seeder” animals are brought in contact with susceptible animals, not for a short period, but for a period similar to what happens in the field. The contact duration should ideally cover the entire infectious period of the seeder animals to make sure that all possible transmission can occur. In pigs, several such transmission experiments have been performed (Table 1). For mathematical animal disease models, information on the transmission rate parameter β and reproduction ratio R is very important. Up to now the methodology of determining these parameters has been used on a very limited scale, and therefore it is important to describe the methodology.

**Table 1:** Summary of within-pen and between-pen transmission experiments with pigs.

**Type of transmission**	**Type of study (number of replicates)**	**Virus strain**	**Vaccine**	**Infection of source first infected pigs**	**Vaccination moment**	**β (95% CI)**	***R* (95% CI)**	**Method**	**References**
Within-pen	5I 5S (1)^a^	O/TAW/97	not used	ID	-		∞ (0.67–∞)	FS	(26)
Within-pen	5I 5S (4)	O/TAW/97	not used	ID	-	6.1 (3.8–10)		GLM	(27)
Within-pen	5I 5S (4)	O/TAW/97	not used	ID	-		∞ (2.4–∞)	FS	(30)^b^
						6.1 (3.7–10)	40 (21–74)	GLM	
Within-pen	1I 1S (5)	O/NET/2001	not used	ID	-		∞ (1.2–∞)	FS	(31)
Within-pen	5I 5S (2)	O/NET/2001	not used	CE	-		∞ (1.3–∞)	FS	(31)
						6.8 (3.2–14.8)		GLM	(31)
						4.4 (2.1–8.4)	23 (11–47)	GLM	(33)^c^
Within-pen	1I 5S (1)	O/SKR/2002	Not used	ID	-	2.1 (0.70–6.1)	7.4 (1.8–30)	GLM	(38)
Within-pen	2I 4S (1)	O/JPN/2010	not used	ID	-	1.3 (0.46–3.5)	3.6 (1.0–13)	GLM	(34)^d^
Within-pen (Feral swine to domestic or feral swine)	2I 4S and 2I 5S	A_24_Cruzeiro	Not used	ID	-	73 (0-∞)	470 (0-∞)	GLM	(39)
Within-pen (Domestic swine to feral swine)	2I 4S (1)	A_24_Cruzeiro	Not used	ID	-	2.3 (0.84–6.2)	15 (4.9–44)	GLM	(39)
Within-pen	5I 5S (1)	O/TAW/97	O/TAW/97	ID	−7 dpi		∞ (0.67–∞)	FS	(26)
Within-pen	5I 5S (2)	O/TAW/97	O/TAW/97	ID	−7 dpi		∞ (1.5-∞)	FS	(30)^b^
						2.0 (1.0–4.0)	11 (4.9–24)	GLM	
Within-pen	5I 5S (1)	O/TAW/97	O/TAW/97^e^	ID	−7 dpi		1.2 (0.2–5.4)	FS	(30)^b^
						0.4 (0.1–1.4)	1.0 (0.1–7.8)	GLM	
Within-pen	5I 5S (2)	O/NET/2001	O Manisa	CE	−14 dpi		2.4 (0.9–6.9)	FS	(31)
						0.66 (0.24–1.8)		GLM	(31)
						0.81 (0.39–1.5)	4.4 (2.1–8.2)	GLM	(33)^d^
Between-pen 0 cm	5I 5S (1)	O/TAW/97	not used	ID	-	0.59 (0.083–4.2)		GLM	(27)
Between-pen 0 cm	5I 4S (2)	O/NET/2001	not used	CE	-	0.14 (0.044–0.33)	1.1 (0.34–2.6)	GLM	(33)
Between-pen 40–70 cm	5I 4S (2)	O/NET/2001	not used	CE	-	0.0 (0.0–0.039)	0.0 (0.0–0.08)	GLM	(33)
Between-pen 0 cm	5I 4S (2)	O/NET/2001	O Manisa	CE	−14 dpi	0.0 (0.0–0.075)	0.0 (0.0–0.35)	GLM	(33)

*β is the transmission rate parameter, i.e., the average number new infections per infectious animal per day, R is the transmission ratio, i.e., the average number of new infections per infectious animal during its entire infectious period. ID, Intra-dermal (injection); CE, contact-exposed; dpi, days post-inoculation or infection; FS, Final Size; GLM, General Linear Model*.

*Only experiments with homogeneous groups of pigs are included [so excluding one pair-wise experiment of Orsel et al. (31) with non-vaccinated source pigs + vaccinated contact pigs) and experiments which lasted long enough to reach the end of the infection chain [so excluding experiments with short exposure times, such as Alexandersen and Donaldson (40)]. From the included experiments, β and R can be estimated*.

a *5I 5S (2) indicates five infected pigs, five susceptible pigs and two replicates*.

b *Meta-analysis of experiments from (26–29)*.

c *Re-calculated from Orsel et al. (31) by GLM method with changes in the model assumptions*.

d *Calculated by GLM method*.

e *Pigs were vaccinated with four times the normal dose*.

In this paper the statistical methods to quantify transmission in an experimental setting are presented. The application of methodology is shown by presenting and discussing articles previously published by the authors and some additional selected contributions where pigs were sampled on a daily basis.

## Materials

To show how statistical methods to quantify transmission parameters can be used, we conveniently selected papers where FMDV transmission in pigs was studied. Many of the studies were from our own group in which mostly the reproduction ratio, transmission rate and infectious period had already been analyzed, but we also identified three additional papers that presented data that could be used for analysis.

The raw data from all papers were extracted. Information on author, interval of the observations, distance between pigs, vaccination status, type of pig as source of infection as well as the recipient, FMDV strain, number of infectious, susceptible and cases as well as the total number of animals were recorded (see Supplementary Material: art transmission in pigs Supplementary File 2.csv). These data were used in a meta-analysis using the GLM method (see below) to calculate the transmission rate parameter β. For the analysis of the infectious period information on author, inoculation route, vaccination status, type of pig strain, pig identification duration of excretion and whether or not the data were censored was recorded (see Supplementary Material: art transmission in pigs time supplementary file 3.csv). The duration of the virus excretion was calculated using exponential survival analysis (41). The analysis was performed in R (42) (see Supplementary Material: art transmission in pigs supplementary file 1.r).

## Methods

### Biology of Transmission of FMDV in Pigs

**Figure 1:**
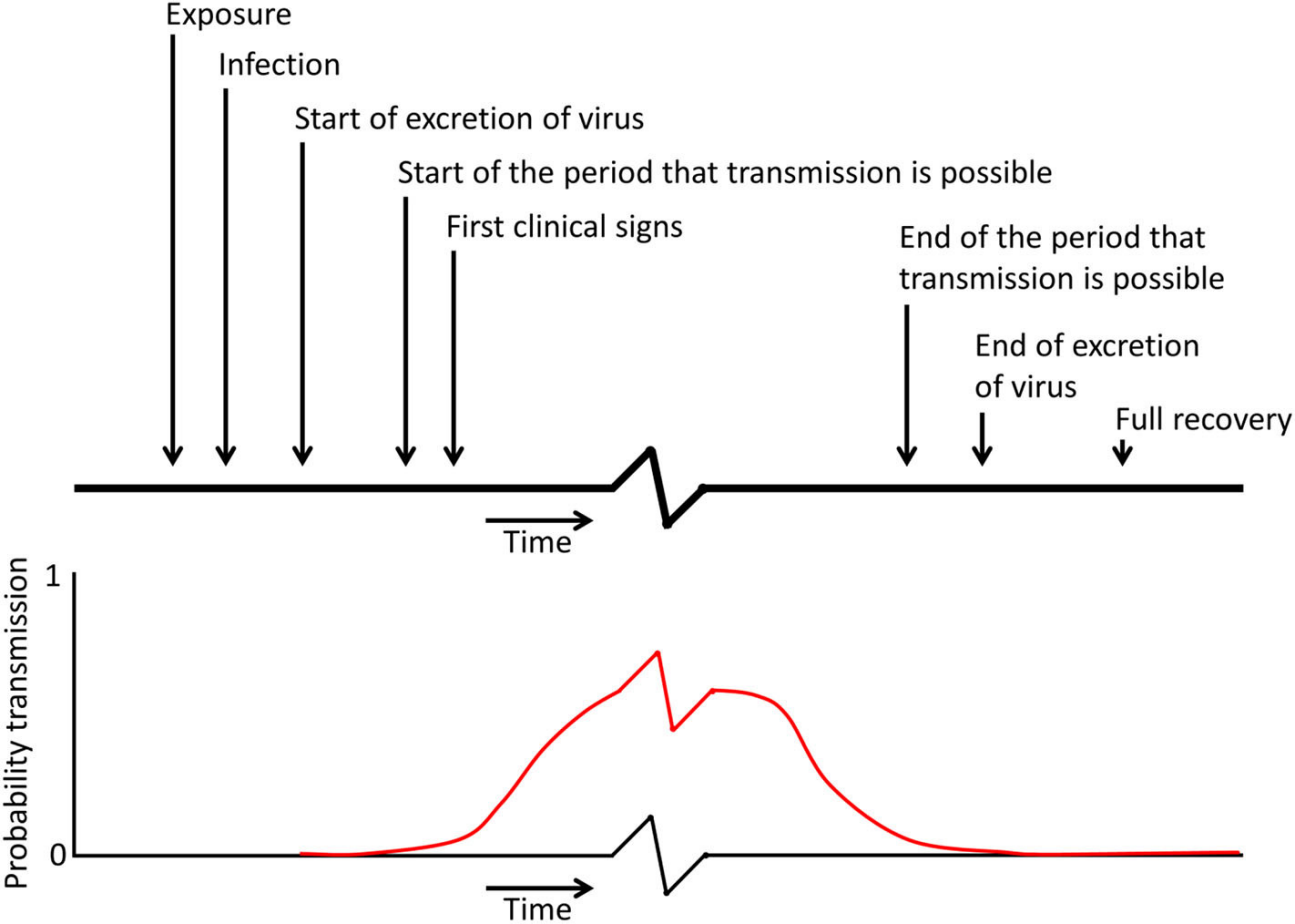
Schematic representation of the different moments in time during infection, which can be used in models to describe the infection process (the x axis and the red curve showing the probability of transmission contain a break as the period in which transmission can occur is probably relatively longer than the other periods indicated on the top of the figure).

The infection process may be described as a sequence of events as depicted in Figure 1. At the very start, susceptible animals become exposed to the presence of one or more seeder animals (Exposure). An animal remains susceptible (S) as long as it has not yet become infected. After exposure to virus it takes time before the virus has sufficiently replicated and is excreted and the animal becomes infectious, and during this latent period an animal is often referred to as exposed (E) or latently infected. During the subsequent infectious period the animal is labeled infectious (I). In infection experiments the time of exposure is often well-established, but the exact time that virus replication and the start of secretion is difficult to measure as there are often only one or two observations per day. Orsel et al. (43) estimated the contribution of transmission before clinical signs were observed. In contrast to cattle the contribution to transmission during the incubation period was very high in pigs; the point estimate for the number of new infections per infectious individual in a completely susceptible population during the incubation period was 13 for non-vaccinated pigs and 1.3 for vaccinated pigs (43). These findings were confirmed by Stenfeldt et al. (44) who observed that the transmission period started ~1 day before clinical signs were observed; even when pigs were only exposed to infectious pigs during a limited 8 h period. In cattle it is assumed that aerosolised FMDV is responsible for infection of cattle, because cattle are highly susceptible for infection via the airborne route (45, 46). Pigs, however, are relatively resistant to infection by natural aerosols (47). The oral route in pigs is also unlikely as the virus will not survive the low pH in the stomach, although infection can occur through exposure of the oropharyngeal tonsil (48). In swine vesicular disease, a disease caused by a different picornavirus but producing clinical disease similar to FMD, infection through the skin is considered the most important route for infection and fighting between pigs can enhance transmission (49). It is not unlikely that in the case of FMDV the skin is also an important port of entry for the virus, but pathogenesis studies after exposure to an FMDV contaminated environment to verify this hypothesis have not been performed.

In pigs the interval between exposure and infection is very short. In cattle newly formed virus is observed within a few hours; the growth rate within the individual host depended on the initial infection dose (50). In the analysis below it is assumed that it is known which pigs are susceptible (S that is not infected), exposed (E), infectious (I), or recovered (R) during the experiment. In many other infections the moment of infection is difficult to estimate, as the time between infection and start of virus shedding is often long and variable. In the FMDV transmission studies discussed in this paper the latent period of infected pigs was ignored. This was based on the observation that virus isolation from oropharyngeal swabs was positive within 24 h in more than 20% of the pigs inoculated by injection in the bulb of the heel. In pigs that were exposed to infectious pigs, a higher proportion was positive in oropharyngeal swabs within 24 h. Therefore, in most experiments, we can conclude that a latent period of <1 day was observed; with only daily observations, inclusion of such a short latent period in the model is not useful. In the studies pigs were classified as being susceptible (S), infectious (I) based on virus isolation from oropharyngeal swabs, not based on clinical signs. All pigs were free of FMD virus and antibodies to the virus before the experiment started.

### Statistical Methods to Estimate Transmission of FMDV in Pigs

To estimate transmission repeated observations on all individuals in an isolated group are needed. At each observation all individuals are classified, based on samples taken at the observation moment and analyzed later, as either susceptible (S), exposed (E), infectious (I), or recovered/removed (R). Furthermore, each of these individuals may have multiple other characteristics that may or may not influence transmission. These characteristics can for example be: vaccinated or not vaccinated, being in the same pen or in a neighboring pen, being in the same pen at an earlier moment (1, 2, 3, …, days ago) etc.

The reproduction ratio R can also be estimated when only the number of susceptible, exposed, infectious and recovered animals at the start and at the end of the experiment are known. This is possible, provided that the experiment lasted long enough to reach the end of the outbreak and is called the estimation of R based on the final size of the outbreak (FS). It is based on the fact that for small numbers of individuals per group the likelihood of the observed outcome of the experiment can be (exactly) calculated for each possible value of the reproduction ratio R. The reproduction ratio that yields the maximum likelihood is then selected (51). The FS estimation of R is independent of the presence of a latent period (52) and can be carried out for any assumed distribution of the infectious period [see Formula 1 in (52) based on (53)].

#### Final Size (FS) Method: Homogeneous Groups

Given any number of susceptible (S > 0) and infectious (I > 0) individuals at the start of the experiment, two events can happen: (i) either an infection occurs and thus the number of susceptible individuals decreases by 1 and the number of infectious individuals (or exposed) increases by 1, or (ii) an infectious individual recovers and the number of infectious individuals decreases by 1 and the number of recovered individuals increases by 1. When an exposed individual does not become infectious immediately, but after a certain lag time (latent period), it is considered E. For sake of simplicity we are assuming no latent period in the formulas used here, so we assume a SIR model; however for SEIR models the same formulas apply (53). In the case of FMDV infection in pigs, virus secretion often starts within the observation period (sampling interval 1 day), so the assumption of no latent period is then valid (see section on Biology above). The alternative event is a recovery of the infectious animal and thus the number of infectious individuals decreases by 1. In formulas:

(1) $$\begin{array}{r} \left(S,I\right)\to \left(S-1,I+1\right)\text{with rate }\beta \frac{\text{SI}}{\text{N}} \end{array}$$

(2) $$\begin{array}{r} \left(S,I\right)\to \left(S,I-1\right)\text{with rate }\alpha I \end{array}$$

with N being the total number of individuals, β is the transmission rate parameter (see definition above) and α is the recovery rate parameter of the infectious individuals, i.e., the average number of infectious individuals that recover (or die) per unit of time, and do not contribute to transmission anymore. This recovery rate parameter is equal to 1/infectious period. Clearly there is a third possibility and that is that no infection and no recovery occurs; i.e., the status stays the same. However, conditional on the fact that something has happened, only these two events can have occurred and from the rates above, it follows that the probability of the first event being an infection event (p) is:

(3) $$\begin{array}{r} p=\frac{\beta \frac{SI}{N}}{\beta \frac{SI}{N}+\alpha I} \end{array}$$

Which simplifies by $R=\frac{\beta }{\alpha }$ and provided I ≠ 0 to:

(4) $$\begin{array}{r} p=\frac{R\frac{S}{N}}{R\frac{S}{N}+1}=\frac{RS}{RS+N} \end{array}$$

For example, in an experiment with pairs, i.e., with only one infectious individual and one susceptible individual (i.e., *S* = 1 and *N* = 2), the probability (p) that the contact individual becomes infected before the infectious animal recovers is:

(5) $$\begin{array}{r} p=\frac{R}{R+2} \end{array}$$

and thus, from observing n pairs with k infections in the contacts, the maximum likelihood estimator (MLE) for R is:

(6) $$\begin{array}{r} \hat{R}=\frac{2k}{n-k} \end{array}$$

For example, when observing 15 contact infections in 30 pairs, $\hat{R}=\frac{30}{15}=2$. For any experiment with more individuals it is necessary to calculate the final size distribution with a computer algorithm (51) which can also accommodate the case where the infectious period has a different distribution than the exponential distribution (52, 53).

#### Generalized Linear Model (GLM) Method: Homogeneous Groups

The GLM method can be used when individuals are sampled at regular time intervals during the experiment (yielding interval data). For the ease of explanation, it is first assumed that all individuals are identical (homogeneous group) in all characteristics except for being susceptible or infectious. The SIR model will be used in which each individual moves from Susceptible to Infectious and then to Recovered. Thus, exposed individuals are ignored. Also ignored are infectious individuals which can recover after some time and become susceptible again (they are included in SIS or SIRS models).

To estimate the transmission rate parameter, the number of susceptible individuals (S) and the number of infectious individuals (I) are counted both at the start and at the end of every time interval, and the length of the interval is determined as Δt. With that information for subsequent intervals during the experiment the transmission rate parameter β can be estimated. Those individuals that are S at the start of the interval and I at the end of the interval are the cases (i.e., new infections), counted as C.

The following relationship between the observed counts and the transmission parameter can be considered as a good starting point for analysis (16):

(7) $$\begin{array}{r} c_{t}=\beta \frac{S_{t}I_{t}}{N_{t}}\Delta t \end{array}$$

where C_t_ is the number of observed cases in the interval [t, t+Δt], β the transmission rate parameter (see definition above), S_t_, I_t_ and N_t_ respectively the number of susceptible, infectious and the total number of individuals at the start of the interval [t, t+Δt] and Δt is the length of the interval. The total number of new cases in interval [t, t+Δt] will be 0, 1, 2, …, with maximum value St (all available susceptibles). In an approximation in which the contribution of new cases to the infectivity occurring during the interval [t, t+Δt] is neglected, the fate of each susceptible individual is independent and identically distributed according to a Bernoulli distribution with infection probability p_t_:

(8) $$\begin{array}{r} p_{t}=1-e^{-\beta \frac{I_{t}}{N_{t}}\Delta t} \end{array}$$

Thus, each susceptible individuals' infection status is independent and identically distributed with values (1) for individuals infected and (0) for individuals not infected at the end of the interval. For all susceptible individuals together there are C_t_ (with value 0, 1, 2, …S_t_) new cases observed; thus binomially distributed with parameters S_t_ and p_t_ as given by the equation above. This binomial distribution follows from the assumption of independent and identically distributed Bernoulli variables.

From the observed C_t_ and the observed I_t_, S_t_ and N_t_, the transmission rate parameter β can now be estimated. For that, a statistical technique called Generalized Linear Models (GLM) is used (54). In that method a function has to be specified that links the expected value of our observed result variable (here C_t_/S_t_) to a linear function of the explanatory variables (here I_t_ and N_t_), and a distribution has to be specified around this expected value. That distribution is needed to minimize the variance around the expected values based on estimated coefficients. The distribution that is used here is the binomial distribution, with S_t_ as the binomial total. As a link function the complementary-log-log function is used.

The expected number of cases per susceptible $\varepsilon \frac{{\text{C}}_{\text{t}}}{{\text{S}}_{\text{t}}}$ is p_t_, (the expectation is indicated by ε) which then gives:

(9) $$\begin{array}{l} \text{cloglog }(\;\varepsilon \frac{{\text{C}}_{\text{t}}}{{\text{S}}_{\text{t}}})=\log\;\left(-\log\left(1-\left(1-e^{-\beta \frac{I_{t}}{N_{t}}\Delta t}\right)\right)\right)\\ =\log(\beta )+\log\;(\frac{I_{t}}{N_{t}}\Delta t) \end{array}$$

Where $\log\left(\frac{I_{t}}{N_{t}}\Delta t\right)$ is the offset and log(β) is the intercept (regression coefficient) estimated in the statistical analysis. The offset is a value that is subtracted from the transformed result variable before fitting the model. In this homogeneous case the model has only one unknown regression coefficient that has to be estimated [log(β)], as there are no differences in susceptibility or infectivity between the individuals. Note that differences in the infectious period distribution (see above in the FS model) do not play a role in the GLM analysis as we observe whether or not the animal is infectious at the beginning (and at the end) of each interval. We assume that the effect that some animals stop virus excretion during the interval can be ignored.

#### Generalized Linear Model (GLM) Method: Heterogeneous Groups

In the studies reviewed, heterogeneity was observed because of vaccination (26, 31) and/or because of spatial separation of animals (33). In the example given in this paper, groups containing vaccinated and non-vaccinated individuals (mixed) are used, but the same formulas can be applied to other settings with heterogeneous groups. Vaccination can have two effects on transmission, and thus on the transmission parameter, i.e., it can affect the susceptibility and the infectivity. The susceptibility effect is that vaccinated individuals may under the same circumstances have a lower (or at least different) probability of becoming infected than non-vaccinated individuals. The infectivity effect of vaccination is that the amount of virus and/or the duration of viral shedding is reduced, and thereby reducing the probability of causing infection (32). Note that any individual that is in a group where there are more infectious individuals present than in a comparison group, will get infected more often compared to the individuals in the comparison group. This is the indirect effect of heterogeneity (55). In groups with both vaccinated and non-vaccinated animals both effects of vaccination on susceptibility and infectivity can be estimated.

The observations in homogeneous groups consisted of I_t_, S_t_, and C_t_. This now changes to I_u,t_, S_u,t_, C_u,t_, I_v,t_, S_v,t_, and C_v,t_ where u stands for non-vaccinated and v stands for vaccinated individuals. The result variable in the analysis is now either $\frac{C_{u,t}}{S_{u,t}}$ or $\frac{C_{v,t}}{S_{v,t}}$ corresponding to infections occurring in non-vaccinated and vaccinated individuals respectively. An indicator for vaccination, the dummy variable IndV with value 1 for vaccinated and 0 for non-vaccinated susceptibles, is introduced as explanatory variable to distinguish the estimate of transmission rate parameter for these two situations with the different recipients. The regression coefficient before the explanatory variable IndV, in this case, represents the effect of vaccination on susceptibility [i.e., the relative susceptibility of a vaccinated susceptible compared to that of a non-vaccinated susceptible (set to 1)].

The offset in the statistical model for the heterogeneous situation uses the total number of infectious individuals, i.e., the offset is now: $\log\left(\frac{I_{u,t}+I_{v,t}}{N_{t}}\Delta t\right)$. The explanatory variable used to estimate the effect of vaccination on infectivity is the fraction of vaccinated infectious individuals: $FrI_{v}=\frac{I_{v,t}}{I_{u,t}+I_{v,t}}$ and thus the regression coefficient before this explanatory variable represents the effect of vaccination on infectivity.

To illustrate this, the transmission rate parameter (β) can be written as the product of the overall contact rate (c), the relative susceptibility of vaccinated individuals (γ_v_) and the relative infectivity of vaccinated individuals (φ_v_). Then the transmission rate parameter for the transmission between vaccinated individuals is β_vv_ = c γ_v_ φ_v_ and between non-vaccinated individuals is β_uu_ = c γ_u_ φ_u_ = c (as the relative susceptibility γ_u_ and infectivity φ_u_ for non-vaccinated individuals is set to 1, being only interested in the relative effects). The transmission rate parameter from vaccinated to non-vaccinated is β_vu_ = c γ_u_ φ_v_ = c φ_v_.

Now one can write the link function equation and identify the regression coefficients that have to be estimated:

$$\begin{array}{l} \text{cloglog }(\;\varepsilon \frac{{\text{C}}_{\text{u or v, t}}}{{\text{S}}_{\text{u or v,t}}})=\log\;\left(-\log\left(1-\left(1-e^{-\beta \frac{I_{t}}{N_{t}}\Delta t}\right)\right)\right)\\ =\log(c)+\log(\gamma_{v})\cdot IndV+\log(\varphi_{v})\cdot FrI_{v}+\log\;(\frac{I_{u,t}+I_{v,t}}{N_{t}}\Delta t) \end{array}$$

The regression coefficients from the analysis are identified by their explanatory variables, thus, these coefficients C0, C1 and C2 are:

C0 (intercept) = log(c)

C1 = log(γ_v_)

C2 = log(φ_v_)

Offset = log$\left(\frac{I_{u,t}+I_{v,t}}{N_{T}}\Delta t\right)$

β = c · γ_v_^IndV^ · ${\text{φ}}_{\text{v}}^{\text{Fr}{\text{I}}_{\text{v}}}$

And thus, the four possible values of the transmission rate parameter are:

From non-vaccinated to non-vaccinated: FrI_v_ =0 and IndV = 0 then β_uu_ = c = e^C0^

From non-vaccinated to vaccinated: FrI_v_ =0 and IndV = 1 then β_uu_ = c · γ_v_ = e^C0+C1^

From vaccinated to non-vaccinated: FrI_v_ =1 and IndV = 0 then β_uu_ = c · φ_v_ = e^C0+C2^

From vaccinated to vaccinated: FrI_v_ =1 and IndV = 1 then β_uu_ = c · γ_v_ · φ_v_ = e^C0+C1+C2^

Note 1: The link function in the heterogeneous case contains an approximation because in the SIR model the average infectivity is measured by an arithmetic average and in the statistical analysis a geometric average is assumed as a linear model on the log scale was used (56). This approximation causes a small error that can be corrected. As both approximations lead to underestimation of the effects on infectivity and susceptibility it can be chosen to just ignore this small error. For a discussion about these errors and the methods to correct them, see (57).

Note 2: In case where the vaccinated and non-vaccinated individuals do not mix, the effect of vaccination on susceptibility and infectivity is completely confounded as both dummy explanatory variables (FrI_v_ and IndV) have value 1 for the vaccinated group and value 0 for the non-vaccinated group. Fitting will give us the effect of vaccination on the transmission rate parameter but whether the effect is on susceptibility or infectivity cannot be inferred.

### Results of Transmission Studies in Pigs

Of the nine papers used in the analysis six papers were from our own group, the three additional papers described an FMDV transmission study in pigs in sufficient detail to calculate the transmission rate parameter and reproduction ratio; we used the GLM method. In a few cases the results were analyzed by both the GLM as well as the final size method.

In total 14 experiments were identified in which pigs were infected with one of five different FMDV strains (O/TAW/97 *n* = 7, O/JPN/2017 *n* = 1, O/NET/2001 *n* = 4, O/SKR/2002 *n* = 1 and A_24_Cruzeiro *n* = 1). In two out of 14 experiments there was physical separation between the pigs and in three experiments the pigs were vaccinated. Table 1 gives an overview of the experimental transmission studies in pigs included in this review. First, results will be reported of the studies in which transmission was studied within a pen, without vaccination as well with homologous and heterologous vaccination. Next, results will be reported of studies on transmission between pens. These latter results are also relevant for transmission between farms, which itself cannot be studied in an experimental setting.

#### Within-Pen Transmission

The first FMDV transmission experiment, discussed here, was performed with three homogeneous groups of pigs (i.e., two groups of pigs that were vaccinated and one group of pigs that was not vaccinated) (26). Each group was housed in a different stable. This experiment was performed using FMDV strain O/TAW/97 for infection, where the effect of vaccination with homologous FMDV O/TAW/97 vaccine 7 and 14 days prior to exposure was tested (−7 dpi and −14 dpi; dpi = days post-inoculation). As control a group of non-vaccinated pigs was included (29). In each group of ten pigs five were inoculated with FMD virus by intradermal injection in the bulb of the heel and mixed with the remaining five contact pigs. The estimated R for the non-vaccinated controls, based on the FS method, was ∞, with 95% confidence interval 0.67-∞ (see Table 1). The wide confidence interval is due to the low number of replicates (only five contact animals in one replicate) and due to the relatively crude estimation method, based on FS only. In a subsequent meta-analysis R was also estimated using the GLM method (30). No significant effect was found for the −7 dpi vaccination compared to the control treatment (also the amount of virus shedding was similar; data not shown here). Vaccination 14 days prior to infection, however, did not result in infectious (vaccinated) pigs, due to complete protection to the injected challenge virus.

In another experiment Eblé et al. (27) studied within-pen and between-pen transmission of FMDV strain O/TAW/97 among non-vaccinated pigs. The within-pen transmission experiment was performed in four replicates of 5S + 5I pigs. Pigs were inoculated by intradermal injection in the bulb of the heel. The estimated transmission rate parameter β was 6.14 per day (see Table 1) (27). Although a value for R was not given in the paper, it can be estimated using β × T, where T is the duration of the infectious period of the inoculated pigs (I pigs). With T roughly estimated as 5 days (from data in the paper), R is ~30 for the within-pen transmission of non-vaccinated pigs according to this study.

In a meta-analysis, in which the above experiments were re-analyzed together with unpublished transmission data (26–30), the GLM method was used. In the GLM method daily data on virus detection in oropharyngeal swabs are used as indicator for infectivity (number of infectious pigs) and infection (number of cases). Based on the combination of the results the total number of non-vaccinated controls now consisted of four replicates of 5S + 5I pigs, the −7dpi vaccination treatment consisted of two replicates of 5S + 5I, a new −7dpi vaccination treatment with a four-fold vaccine doses consisted of one replicate of 5S + 5I, the −14 dpi treatment with a homologous vaccine (O/TAW/97) consisted of two replicates of 5S + 5I, and a new −14 dpi treatment with a heterologous vaccine (O Manisa) consisted of one replicate of 5S + 5I. In the experiments included in the meta-analysis the pigs were always inoculated by intradermal injection in the bulb of the heel. The corresponding β and R values can be found in Table 1. This resulted in a point estimate of R of 40 for non-vaccinated pigs and of a significantly lower value of 11 for pigs vaccinated at−7 dpi. A four-fold higher vaccine dose, also at −7 dpi, reduced the *R*-value (from 11) to a significantly lower value of 1.0, but not significantly below 1 (30).

Orsel et al. (31) studied the effect of vaccination on susceptibility of contact pigs in pair-wise transmission experiments using FMD virus strain O/NET/2001. In the pair-wise transmission experiments non-vaccinated and vaccinated contact pigs (S) were exposed to non-vaccinated seeder pigs (I) inoculated by intradermal injection in the bulb of the heel. The experiment was performed with six replicates of a pair-wise experiment with 1S + 1I pig for both the vaccinated and the non-vaccination contacts. Some of the inoculated pigs did not become infectious. The FS R estimate for transmission between non-vaccinated pigs was ∞ (1.2-∞) (see Table 1), not different from the value between 30 and 40 found earlier (28, 30). In the pair-wise experiment using vaccinated contact pigs four inoculated pigs became infectious and transmitted FMDV to all vaccinated contacts (31); this indicated that vaccination did not reduce susceptibility.

Since no significant differences were observed between susceptibility in vaccinated or non-vaccinated pigs, Orsel et al. (31) performed a second experiment in which the effect of vaccination on both susceptibility and infectivity was studied. Due to the fact no infection was seen in the inoculated pigs (26, 28) that had been vaccinated 14 days prior to infection, Orsel et al. (31) changed the needle infection to challenge by contact to non-vaccinated needle infected pigs. The experiments were performed with two homogenous groups of vaccinated or non-vaccinated pigs. Vaccinated pigs (−14 dpi) were exposed to FMDV by housing them together with non-vaccinated seeder pigs. These vaccinated pigs became infectious by this route of infection and shed virus in oropharyngeal fluid for several days. After infection the non-vaccinated and (−14 dpi) vaccinated pigs that were infected by contact exposure were brought into contact with respectively non-vaccinated and (−14 dpi) vaccinated contact pigs. As the objective of that study was the estimation of R within a group of vaccinated pigs, the data on transmission from non-vaccinated seeders to vaccinated contact pigs were not included in the original publication. But based on the original data and the GLM analysis explained earlier we now also calculated an R of 23 (95% CI 11–47) for non-vaccinated pigs and an R of 4.4 (95%CI 2.1–8.2) for vaccinated pigs. This indicates that vaccination reduces transmission significantly, but the estimate for within-pen transmission for vaccinated pigs was not below 1 (Table 1).

We identified three studies where transmission between pigs was studied (Table 1), but that did not analyse the transmission rate parameter or R. The first study was performed with O/SKR/2002 (38), and yielded an R of 7.4 (95%CI 1.8–30). The second one was performed with O/JPN/2010 (34), this yielded an R of 3.6 (95%CI 1.0–13). The third study (39) was interesting as different types of pigs, feral and domestic, were used as infectious and contact pigs. The GLM model using type of source pig as additional explanatory variable fitted significantly better to the results, i.e., lower Akaikes Information Criterion (14 for the null-model and nine for the model with source as explanatory variable) (58, 59). Analysis of this study alone indicates that feral pigs are more infectious than domestic pigs.

#### Between-Pen Transmission

Pen-to-adjacent-pen transmission experiments of FMDV in non-vaccinated pigs have been described (27, 40, 46). In the first experiments (27, 40) the total number of contact pens in the studies was four, which is very limited for quantification of between-pen transmission. van Roermund et al. (33) therefore performed three pen-to-pen transmission experiments, where the number of contact pens (cumulated over all replicates) was eight per experiment.

Alexandersen and Donaldson (40) did not observe pen-to-adjacent-pen transmission from non-vaccinated donor pigs to non-vaccinated receiver pigs in any of the four replicates where the exposure time was 24–48 h (47). The only possible transmission route in this experiment was the airborne route. Eblé et al. (27) studied within-pen and between-pen transmission of FMDV strain O/TAW/97 among non-vaccinated pigs. The between-pen transmission experiment was performed in one replicate of 5S + 5I pigs, in which the S and I pigs were separated by a single wall. The I pigs were inoculated by intradermal injection in the bulb of the heel. The estimated between-pen transmission rate parameter β was 0.59 per day, which was significantly lower than the within-pen transmission rate parameter of 6.14 per day (see Table 1) (27). The expected time to infection of the first pig in the adjacent pen was estimated at 16 h, much longer than that of the first contact pig within the pen, which was estimated 1.6 h (derived from the estimated β; experimental observations were done on a daily basis, not more frequent). van Roermund et al. (33) performed three pen-to-pen transmission experiments with two replicates. Each replicate consisted of five seeder pigs housed in a central pen surrounded by four separate pens, each containing one contact pig. The FMD virus strain used was O/NET/2001. The seeder pigs in the central pen were infected by contact exposure to needle inoculated seeders. The exposure of the pigs in the adjacent pens was thus to already contact-infected pigs, a more natural infection route than needle infection, this method had been used in a previous direct transmission experiment (31). All pen walls in the experiments consisted of solid barriers ~1.2 m high that were not glued or cemented to each other or to the floor. In the first experiments, all non-vaccinated contact pigs were housed in pens which were separated by a walkway of 40–70 cm from the pen with the seeder pigs (so two solid barriers between contact and seeder pigs). In the second experiment, all non-vaccinated pigs were housed in pens that were adjacent, only separated by one solid barrier from the pen with the seeder pigs. In the third experiment, the set-up of the second experiment was repeated but with all pigs vaccinated (−14 dpi). The between-pen transmission events per experiment were analyzed as eight replicates (2 × 4 pens) of 1S + 5I pigs. In non-vaccinated pigs, no transmission occurred when the central pen with five seeder pigs and the pens with contact pigs were separated by a 40–70 cm wide walkway (so two solid barriers). The seeder pigs in the central pen, however, were excreting FMD virus in the oropharyngeal fluid for 8 days and virus could be isolated from air above the pen. The estimated between-separated-pen R was 0 (0–0.08). This observed R was significantly below 1 (see Table 1).

In the second experiment using non-vaccinated pigs in adjacent pens, transmission was observed from seeder pigs to contact pigs. In four out of eight individually housed contact pigs FMDV infection was detected, in all cases at 3 days post-exposure. The corresponding between-adjacent-pen R was estimated 1.1 (0.34–2.56) which is not significantly above or below 1 (Table 1). When the second experiment was repeated with pigs that were vaccinated 14 days prior to exposure, no transmission was observed to the adjacent pens. The estimated between-adjacent-pen R for vaccinated pigs was 0 (0–0.35), which is significantly below 1 and thus pen-to-adjacent-pen transmission was stopped after vaccination of pigs. Vaccination alone or separation alone did not reduce R significantly below 1, so the combined effect of separation and vaccination is effective to reduce transmission.

#### Meta-Analysis

The data are given in Supplementary Material “art transmission in pigs supplementary file 2.csv” and “art transmission in pigs time supplementary file 3.csv.” The analysis is given in Supplementary Material “art transmission in pigs supplementary file 1.r.” Univariate analysis of the GLM models with distance, vaccination, type of source pig, type of recipient pig and strain as possible explanatory variables yielded the lowest AIC (273) with the model including distance, which is a huge difference with the AIC of the null model (without explanatory variable) which had an AIC of 493. Using forward regression analysis vaccination was the explanatory variable with the lowest AIC in the models with two explanatory factors. Most models using three explanatory variables did not converge, we therefore stopped the analysis. The estimated transmission rate parameter β for within-pen transmission of non-vaccinated pigs was 5.2 day^−1^ (95%CI 4.0–6.8), that for vaccinated pigs (14 days prior to infection with heterologous vaccine) was 0.60 day^−1^ (95%CI 0.31–1.1) whilst the estimated β for between-pen transmission (distance 0 cm) of vaccinated pigs was 0.032 day^−1^ (95%CI 0.013–0.082).

The duration of virus excretion (infectious period) was analyzed by exponential survival analysis. The univariate analysis showed a significant effect of vaccination, but contribution was found for inoculation, type of pig or virus strain. The average duration of virus excretion was 7.5 days (95%CI 5.6–10) for non-vaccinated pigs and 6.3 days (95%CI 5.2–7.5) for vaccinated pigs.

Based on the calculated β and duration of excretion we can calculate the R (the confidence interval is calculated under the assumption that β and the duration of excretion are independent). The R for within-pen transmission of non-vaccinated pig is 39 (95%CI 29–59), and 3.7 (95%CI 1.9–7.3) for vaccinated pigs. The R for between-pen transmission of vaccinated pigs is 0.20 (95%CI 0.079–0.52) which is significantly below 1.

## Discussion

The objective of this paper was to give an overview of statistical methods to experimentally quantify transmission; to give an example how these methods can be used, we reviewed papers on experimental FMDV transmission in pigs. We have focussed on the whole transmission chain in pigs. Whilst we are aware that others have analyzed part of the transmission chain (35, 37), although valuable, it does not provide all the information on transmission and was therefore not included in the review. We have limited the review to pig to pig transmission and not included transmission from pigs to other species or vice versa (36).

The results presented show that due to the inherently small scale of the experiments, replications are typically needed to obtain estimates of sufficient quality, i.e., unbiased and with sufficiently small confidence intervals. By combining multiple experiments in one meta-analysis the precision can be improved. For an experiment of a given scale and a given number of replicates, sampling animals at regular time intervals during the experiment, and analyzing the results using the GLM method, allows for a smaller variance in R estimates than if only final size (FS) information is used. In non-vaccinated pigs the within-pen transmission is extremely efficient. The point estimates for R range from 10 to 40 (27, 30). In further transmission studies in pigs two control measures were evaluated: vaccination and physical separation, both separately and in combination.

The reviewed studies show that vaccination 7 days prior to infection with a four-fold vaccine dose resulted in an estimated R close to 1, but not significantly below 1. Thus, even though transmission is reduced significantly by vaccination, within a pen the infection will still spread among vaccinated pigs of −7 dpi (26, 30). Other studies (23, 24) claim full protection in C_1_ Oberbayern and O_1_ Lausanne exposed pigs when vaccinated at −7 dpi, but in these studies the time of exposure to seeder pigs was limited to only 1–4 h. For vaccinated pigs at −14 dpi, in the first studies R could not be estimated as needle inoculation did not result in infected vaccinated animals (26, 28). The study, however, indicated that susceptibility of the vaccinated pigs for needle challenge was absent, which will in many cases prevent introduction on the farm and reduce between-farm transmission. In the pair-wise study by Orsel et al. (31) no reduced susceptibility was observed when pigs were vaccinated 14 prior to contact with non-vaccinated seeder pigs. Because no infected vaccinated pigs were observed in the first experiments using needle challenge, in the consecutive experiments the vaccinated pigs were exposed to non-vaccinated seeder pigs. By using this method, it was possible to obtain infected vaccinated pigs (−14 dpi). Although transmission in the vaccinated pigs was reduced compared to the non-vaccinated pigs, R was still above 1 (31).

In our meta-analysis separation is the strongest single factor that influences the transmission rate parameter. The meta-analysis confirms the previous between-pen transmission studies that separation can significantly reduce both β and R. However, R is not reduced to values below 1, when only one solid barrier is present between pigs. Separation of pigs with two solid barriers and a walkway of 40 to 70 cm in between, or vaccination (−14 dpi) in combination with one solid barrier reduced R significantly to values below 1. Thus, these studies show that vaccination helps to block between-pen transmission. Why within-pen transmission of FMD is so efficient compared to between-pen transmission is not clear. The fact that physical separation of pigs reduces transmission so efficiently suggests that airborne infection in pigs does not play an important role. The fact that pigs are relatively resistant to airborne infection has been confirmed in other studies (35, 47, 60). So direct contact with virus excreted in the environment, as has been shown for cattle (61), may play a more important role in pigs as may infection due to fighting (e.g., for food), where virus from pig or environment can directly be transmitted into small wounds of contact pigs. The study by Mohamed et al. (39) give a similar indication, as feral pigs seem to be more infectious than domestic pigs; they are reported to fight more. The role of animal behavior is also shown by the fact that transmission from cattle to pigs is limited compared to transmission from cattle to cattle (36).

Although the hygiene measures taken in the experiments were probably stricter than the current practice on commercial farms, handling of pigs was more intensive as daily samples were collected, so transmission by handling itself can never be excluded. However, the between-pen transmission experiment with non-vaccinated pigs showed no transmission at all when pens were separated by a 40 to 70 cm wide walkway (so two solid barriers), provide proof that hygienic measures can reduce transmission.

The between-pen transmission studies show that transmission of FMDV between farms will be blocked. Still it has been shown to occur even when animal movement is prohibited during an epidemic (62). The major transmission routes left in case of a stand-still are people and inanimate objects moving between farms, in such a case emergency vaccination will be effective as the studies show that vaccination can help even when separation of pigs is limited.

## Conclusions

In many FMD epidemics, vaccination contributed significantly to the control of FMDV as one of the components of the national control and eradication program (4, 63, 64). Transmission experiments as reviewed in this paper can be used to support the FMD control policy. The methods described in this paper can be used to analyse experimental transmission studies. The experimental transmission studies show that vaccination in combination with physical separation can reduce transmission of FMDV in pigs significantly. A combination of a physical and immune barrier is essential for the control FMDV with respect to between-farm transmission and also for reduction of transmission within a pig farm.

## Data Availability Statement

All datasets generated for this study are included in the article/Supplementary Material.

## Author Contributions

AD, HR, TH, PE, and MJ contributed to the ideas represented in this paper. AD was responsible for the analysis of the data. HR and AD were responsible for the reporting and MJ, TH, and PE for the critical reading. All authors contributed to the article and approved the submitted version.

## Conflict of Interest

The authors declare that the research was conducted in the absence of any commercial or financial relationships that could be construed as a potential conflict of interest.
